# Hospitalisation Is a Strong Risk Factor for Discontinuation of Sodium‐Glucose Cotransporter 2 Inhibitors for Type 2 Diabetes

**DOI:** 10.1111/dom.71219

**Published:** 2026-08-17

**Authors:** Tamara Y. Milder, Leah Shepherd, Hans Kristian Raket, Jerry R. Greenfield, Richard O. Day, Jialing Lin, Juliana de Oliveira Costa, Sophie Stocker, Carol A. Pollock, Brendon Neuen, Min Jun, Jane Ludington, Sallie‐Anne Pearson, Michael O. Falster

**Affiliations:** ^1^ Medicines Intelligence Research Program, School of Population Health University of New South Wales Sydney Australia; ^2^ School of Clinical Medicine, UNSW Medicine & Health, St Vincent's Healthcare Clinical Campus University of New South Wales Sydney Australia; ^3^ St Vincent's & Mater Clinical School, School of Medicine The University of Notre Dame Sydney Australia; ^4^ Department of Endocrinology St Vincent's Hospital Sydney Australia; ^5^ Department of Clinical Pharmacology Copenhagen University Hospital—Bispebjerg and Frederiksberg Copenhagen Denmark; ^6^ Copenhagen Phase IV Unit, Department of Clinical Pharmacology and Center for Clinical Research and Prevention University Hospital of Copenhagen—Bispebjerg and Frederiksberg Copenhagen Denmark; ^7^ Victor Chang Cardiac Research Institute Sydney Australia; ^8^ Department of Clinical Pharmacology and Toxicology St Vincent's Hospital Sydney Australia; ^9^ International Centre for Future Health Systems University of New South Wales Sydney Australia; ^10^ Faculty of Medicine and Health The University of Sydney Australia; ^11^ Department of Pharmacy Westmead Hospital Sydney Australia; ^12^ Department of Renal Medicine Royal North Shore Hospital Sydney Australia; ^13^ The George Institute for Global Health, University of New South Wales Sydney Australia

**Keywords:** DPP‐IV inhibitor, pharmaco‐epidemiology, real‐world evidence, SGLT2 inhibitor, type 2 diabetes

## Abstract

**Aims:**

Sodium–glucose cotransporter 2 inhibitors (SGLT2i) are commonly withheld during hospitalisation because of concerns about diabetic ketoacidosis. We examined whether hospitalisation was associated with discontinuation of SGLT2i and compared patterns with another anti‐hyperglycaemic medicine without similar inpatient safety concerns: dipeptidyl peptidase‐4 inhibitors (DPP‐4i).

**Materials and Methods:**

We conducted a retrospective new‐user cohort study using linked, population‐level data for adult residents of New South Wales, Australia. Adults aged ≥ 40 years initiating SGLT2i or DPP‐4i (separate cohorts) between 2016 and 2020 were followed until death, mid‐2021, or treatment discontinuation (gap of ≥ 90 days without a dispensing of the index medicine). We used Cox proportional hazards models to estimate the association between hospitalisation and discontinuation, adjusting for demographic and clinical characteristics. We treated the ‘hospitalisation period’ (the inpatient stay plus the 90 days following discharge) as a time‐dependent exposure.

**Results:**

Among people initiating SGLT2i (*n* = 106 098), the median age was 63 years and 61% were male. Overall, 35.1% were hospitalised during follow‐up, and discontinuation was substantially more frequent during the hospitalisation period (56.4 per 100 person‐years) compared with other follow‐up times (37.6 per 100 person‐years). This association remained significant after adjustment (HR: 1.83; 95% CI: 1.79–1.87). Similar patterns were observed among DPP‐4i treated people (HR: 1.55; 95% CI: 1.51–1.58).

**Conclusions:**

Hospitalisation is a strong risk factor for discontinuation of SGLT2i and DPP‐4i therapy. Strategies to support re‐initiation of SGLT2i during transitions from hospital to community care are needed to prevent harm and maximise cardiorenal protective effects of these medicines.

## 1 Introduction

Sodium‐glucose cotransporter 2 inhibitors (SGLT2i) are a pillar of therapy for people with type 2 diabetes, heart failure (HF) and chronic kidney disease (CKD) [1]. The continuation of these medicines for people who can tolerate them is critical. Even 30 days without continued empagliflozin use in people with HF is associated with a significant increased risk of cardiovascular death or HF hospitalisation [2].

SGLT2i are often paused in hospital in part because of the rare risk of diabetic ketoacidosis (DKA), with the aim to re‐start therapy once discharged (provided no contraindications) [3]. However, recent evidence suggests that therapy may not be restarted after hospital discharge, with close to 30% of SGLT2i‐treated people with type 2 diabetes hospitalised for HF discontinuing therapy in the 90 days following discharge [4]. Most of these people did not re‐initiate therapy within the year post‐hospitalisation. This is highly concerning as people with HF are at very high risk of adverse outcomes and hospitalisation should be a catalyst for initiation, not discontinuation, of these medicines. A Danish study similarly found one‐third of people initiating SGLT2i who discontinued therapy had a hospitalisation in the 3 months prior to discontinuation, although they did not explicitly test for associations between hospitalisation and discontinuation [5]. These findings raise questions as to whether hospitalisation is disruptive to the use of SGLT2i.

Hospitalisation is a key opportunity to optimise pharmacotherapy yet transitions between care settings is a period of heightened risk of medication errors and miscommunication [6]. We therefore investigated the extent to which hospitalisation was a risk factor for SGLT2i discontinuation. We also examined if similar patterns of discontinuation were observed with another frequently used anti‐hyperglycaemic medicine class that does not have the same safety concerns about continuation during hospitalisation, dipeptidyl peptidase‐4 inhibitors (DPP‐4i).

## 2 Materials and Methods

### 2.1 Study Design

We conducted a retrospective new‐user cohort study using linked, administrative data in New South Wales (NSW), Australia's most populous state (Figure S1).

### 2.2 Setting and Data Sources

In Australia, all citizens and permanent residents are entitled to publicly funded universal healthcare [7]. The hospital sector includes a mixture of public and private hospital facilities, with public hospitals primarily funded and managed by the states and territories, and private hospitals (primarily elective procedures) funded through health insurance funds and patient out‐of‐pocket payments. Subsidised access to prescription medicines is funded by the Australian Government via the Pharmaceutical Benefits Scheme (PBS). People discharged from public hospitals in NSW typically receive approximately 1 week's supply of medicines. People discharged from private hospitals receive a PBS prescription, whereby they can access an initial supply, with potential repeats.

We used data from the Medicines Intelligence Data Platform [8], which contains population‐level linked data for all adults residing in NSW from 2002 to 2022. The platform contains linked data on hospitalisation (public and private hospitals), pharmaceutical dispensing data from the PBS, claims for services through the Medicare Benefits Schedule (MBS), and mortality data from the National Death Index. The Australian Institute of Health and Welfare and the NSW Centre for Health Record Linkage performed the data linkage.

The study was approved by the Australian Institute of Health and Welfare Human Research Ethics Committee (approval number EO2021/1/1233) and NSW Population and Health Services Research Ethics Committee (approval number 2020/ETH02273).

### 2.3 Medicines of Interest

We identified SGLT2i and DPP‐4i based on World Health Organisation Anatomical Therapeutic Chemical (ATC) codes (Table S1), which we mapped to PBS item numbers. During the study period, SGLT2i and DPP‐4i were indicated on the PBS for the treatment of type 2 diabetes as first add‐on anti‐hyperglycaemic therapy to metformin if a patient's glycated haemoglobin (HbA1c) was > 7% (53 mmol/mol). From 2022 onwards (i.e., after the study period), SGLT2i had separate HF and CKD listings that were irrespective of the patient's diabetes status. During the study period, the general co‐payment for SGLT2i and DPP‐4i (amount a person pays towards the cost of a PBS‐subsidised medicine) was approximately A$40 per prescription. Many people on low incomes, for example, all social security recipients, are eligible for a reduced (concessional) co‐payment of about A$7 per prescription.

### 2.4 Study Population

We identified two cohorts of adults, aged 40 years and over, initiating each of the two medicine classes of interest, SGLT2i and DPP‐4i between the start of 2016 and 30 June 2020. We excluded people who had potential linkage errors or missing dates of death or were living outside NSW at the date of initiation. The two cohorts were not mutually exclusive; a person could belong to both cohorts if they initiated both classes of medicines. We defined initiation as the first dispensing of the medicine class within the period, with no dispensing in the 1‐year prior.

### 2.5 Outcome

Our primary outcome was discontinuation of the medicine of interest. As PBS data do not contain information about prescribed daily dosage of medicines, we assumed that each SGLT2i or DPP‐4i dispensing provided a 30 day medicine supply. This assumption was validated by examining the distribution of the time between subsequent dispensing of the same medicine (PBS item code). We allowed for switching between medicines within the same class. We accounted for stockpiling and early refills of the same medicine; if a dispensing occurred before the end of estimated supply of the previous dispensing, we carried over the remaining supply.

We considered a person to have discontinued if they had a period of 60 or more days past the end of estimated supply without another dispensing (i.e., 90 days after the dispensing date). The discontinuation date was set as the last date of estimated supply.

We followed people up to the latest available data (mid‐2021), death or discontinuation, whichever came first. We did not consider re‐initiation and counted only the first discontinuation event.

### 2.6 Exposure

Our key exposure was hospitalisation during the follow‐up period, measured as a time‐dependent exposure. We defined the ‘hospitalisation period’ as the hospital stay (including any transfers between hospitals and changes in types of care as a continuation of a single hospital stay) plus the 90 days following discharge. If a SGLT2i or DPP‐4i was initiated after hospital discharge, we did not count the period following initiation as belonging to a hospitalisation period. The 90 day period was chosen to allow for the use of any existing medicines supply that may have been dispensed prior to hospitalisation and any supply provided from the hospital.

### 2.7 Covariates

We measured sociodemographic information at the time of initiation of the medicine of interest (Figure S1) including age, sex, and concessional beneficial status. We measured geographic remoteness and socioeconomic status based on the geographic area of residence. We classified remoteness according to the Accessibility/Remoteness Index for Australia (ARIA), and socioeconomic status according to the Socio‐Economic Indexes for Areas (SEIFA) Index of Relative Socio‐Economic Disadvantage (IRSD). We also measured year of initiation.

To characterise both the existing medicine burden and identify select morbidities of interest, we examined medicines dispensed on or within the 90 days prior to initiation. We identified people treated for hypertension, hyperlipidaemia, congestive HF, arrhythmia, gout, renal disease, and anxiety and/or depression, as well as use of antiplatelets and anticoagulants using the Rx‐Risk classification [9]. The Rx‐Risk Index is a validated tool that identifies treatment for people's' comorbidities based on their prescription medicine dispensing using ATC codes [9]. The Medicines Intelligence Data Platform does not contain diagnostic codes for comorbidities collected outside of hospitalisations. To characterise prior engagement with the health system, we quantified number of consultations with a general practitioner (GP) and number of hospitalisations in the year prior to initiation.

### 2.8 Statistical Analyses

We used descriptive measures to summarise the characteristics of people at initiation of the medicine of interest. This was done for each cohort (SGLT2i or DPP‐4), and for those with and without a discontinuation episode. Categorical data are presented as frequencies and percentages, while continuous variables are presented as medians and interquartile ranges (IQR).

We used Cox proportional hazards models to estimate hazard ratios (HR) for characteristics associated with treatment discontinuation. Models included covariates for potential confounders of age, sex, remoteness, socioeconomic status, concessional beneficiary status, year of medicine initiation, presence of comorbid conditions, and prior medicine and service use. To test for the potential association between hospitalisation and treatment discontinuation, we created a time‐dependent exposure for the hospitalisation period, beginning on the day of hospitalisation and finishing at 90 days post‐discharge. If a person had a subsequent admission within this period, the exposure continued for 90 days following discharge of the subsequent admission. We included this time‐dependent variable in the Cox proportional hazards models outlined above, in both univariate and multivariate models.

### 2.9 Sensitivity Analysis

We performed a sensitivity analysis to account for people stopping their anti‐hyperglycaemic medicine but ‘switching’ to an alternate anti‐hyperglycaemic medicine with cardiorenal protection. To perform this analysis, we censored a person (rather than count as having discontinued) if they initiated (no dispensing in prior 1 year) specific medicine/s within the period used to define discontinuation. For the SGLT2i cohort, this was a glucagon‐like peptide‐1 receptor agonist (GLP‐1 RA). For the DPP‐4i cohort, this was an SGLT2i or GLP‐1 RA.

We also performed a sensitivity analysis in which we changed the definition of a ‘hospitalisation period’ as the hospital stay plus 30 days, rather than 90 days following discharge. While 90 days was chosen to allow greater confidence in exhaustion of supply (e.g., medicines dispensed prior to admission; supplied in hospital), discontinuation events occurring 2–3 months after a hospital discharge maybe less related to a hospitalisation compared with those occurring within 1 month of discharge.

## 3 Results

### 3.1 People Treated With SGLT2i

We identified 106 098 eligible people aged over 40 initiating an SGLT2i between the start of 2016 and mid‐2020. The median age at initiation was 63 years, and 61% were male (Table 1). Of the cohort, 66 989 (63.1%) discontinued treatment during follow‐up. There was a mean follow‐up period of 1.6 years. Characteristics of those with and without a SGLT2i discontinuation episode are presented in Table S2. A total of 37 226 (35.1%) of SGLT2i‐treated people were hospitalised during follow‐up, corresponding to 9% of total follow‐up time being within the hospitalisation period (Table 2). Discontinuation rates were higher in hospitalisation periods (56.4 per 100 person‐years) than all other times (37.6 per 100 person‐years).

**TABLE 1:** Characteristics of SGLT2i and DPP‐4i cohorts.

Characteristics	SGLT2i cohort	DPP‐4i cohort
	*N* = 106 098	*N* = 91 960
Age, years, median (IQR)	63 (55–71)	66 (57–74)
Age categories, years, *n* (%)		
40–49	13 726 (12.9)	10 686 (11.6)
50–59	27 814 (26.2)	20 222 (22.0)
60–69	35 810 (33.8)	27 260 (29.6)
70–79	23 187 (21.9)	22 372 (24.3)
80+	5561 (5.2)	11 420 (12.4)
Sex, *n* (%)		
Male	64 229 (60.5)	52 574 (57.2)
Female	41 869 (39.5)	39 386 (42.8)
Residence remoteness, *n* (%)		
Major cities	71 893 (67.8)	63 638 (69.2)
Inner regional/outer regional	32 378 (30.5)	26 673 (29.0)
Remote/very remote	1318 (1.2)	1230 (1.3)
Unknown	509 (0.5)	419 (0.5)
Socioeconomic status, *n* (%)		
Q5 (least disadvantaged)	19 377 (18.3)	16 024 (17.4)
Q4	13 626 (12.8)	11 739 (12.8)
Q3	21 957 (20.7)	18 788 (20.4)
Q2	23 474 (22.1)	20 113 (21.9)
Q1 (most disadvantaged)	27 148 (25.6)	24 869 (27.0)
Unknown	516 (0.5)	427 (0.5)
Concessional beneficiaries, *n* (%)	61 528 (58.0)	59 015 (64.2)
Calendar year of SGLT2i/DPP‐4i initiation, *n* (%)		
2016	19 664 (18.5)	21 015 (22.9)
2017	27 344 (25.8)	20 415 (22.2)
2018	24 821 (23.4)	21 662 (23.6)
2019	23 288 (21.9)	20 121 (21.9)
2020	10 981 (10.3)	8747 (9.5)
History of selected morbidities and medicine use^a^, *n* (%):		
Anticoagulants	9878 (9.3)	10 659 (11.6)
Antiplatelets	14 214 (13.4)	12 410 (13.5)
Arrhythmia	4748 (4.5)	5014 (5.5)
Congestive heart failure	10 026 (9.4)	10 638 (11.6)
Hypertension	47 217 (44.5)	41 051 (44.6)
Hyperlipidaemia	81 493 (76.8)	66 942 (72.8)
Gout	9506 (9.0)	9301 (10.1)
Renal disease	352 (0.3)	1333 (1.4)
Anxiety and/or depression	27 380 (25.8)	24 089 (26.2)
Number of unique medicines dispensed in 90 days prior to and including the index date, *n* (%)		
1–4	26 178 (24.7)	24 315 (26.4)
5–9	56 631 (53.4)	45 295 (49.3)
10+	23 289 (22.0)	22 350 (24.3)
Number of hospitalisations in year prior to and including the index date, *n* (%)		
0	67 100 (63.2)	54 575 (59.3)
1	29 234 (27.6)	26 749 (29.1)
> 1	9764 (9.2)	10 636 (11.6)
Number of GP consultations in year prior to index date, *n* (%)		
0–9	59 001 (55.6)	50 107 (54.5)
10–19	35 205 (33.2)	30 297 (32.9)
20+	11 892 (11.2)	11 556 (12.6)

Abbreviation: IQR, interquartile range.

^a^ Estimated using the Rx‐Risk Comorbidity Index.

**TABLE 2:** Discontinuation rates among people treated with SGLT2i or DPP‐4i, overall and stratified by hospitalisation time period.

	SGLT2i cohort	DPP‐4i cohort
Overall follow‐up		
Total person‐years	169 973	141 224
Number discontinued	66 989	58 572
Rate per 100 person‐years	39.4	41.5
During a hospitalisation period		
Total person‐years	16 009	16 319
Number discontinued	9031	7808
Rate per 100 person‐years	56.4	47.8
All other follow‐up times		
Total person‐years	153 964	124 905
Number discontinued	57 958	50 764
Rate per 100 person‐years	37.6	40.6

### 3.2 People Treated With DPP‐4i

We identified 91 960 eligible people initiating DPP‐4i. The median age at initiation was 68 years and 57% were male (Table 1). Of the cohort, 58 572 individuals (63.7%) had a discontinuation episode during follow‐up. There was a mean follow‐up period of 1.5 years. Characteristics of those with and without a DPP‐4i discontinuation episode are presented in Table S3. Of the DPP‐4i‐treated people, 37.3% were hospitalised during follow‐up, with higher rates of discontinuation in the hospitalisation period compared with all other times (47.8 vs. 40.6 per 100 person‐years, respectively) (Table 2).

### 3.3 Association Between All‐Cause Hospitalisation and Treatment Discontinuation

In SGLT2i‐treated people, the adjusted risk of SGLT2i discontinuation was 1.8 times higher (95% CI: 1.79–1.87) in the hospitalisation period compared to other times (Figure 1 and Table S4). The relationship between all‐cause hospitalisation during follow‐up and SGLT2i discontinuation, was stronger than most other characteristics measured, including age (e.g., 60–69 years vs. 40–49 years, HR: 0.83, 95% CI: 0.81–0.85), sex (female HR: 1.17, 95% CI: 1.16–1.19) and polypharmacy (e.g., 10+ medicines vs. 1–4 medicines, HR: 0.72, 95% CI: 0.70–0.75).

**FIGURE 1:**
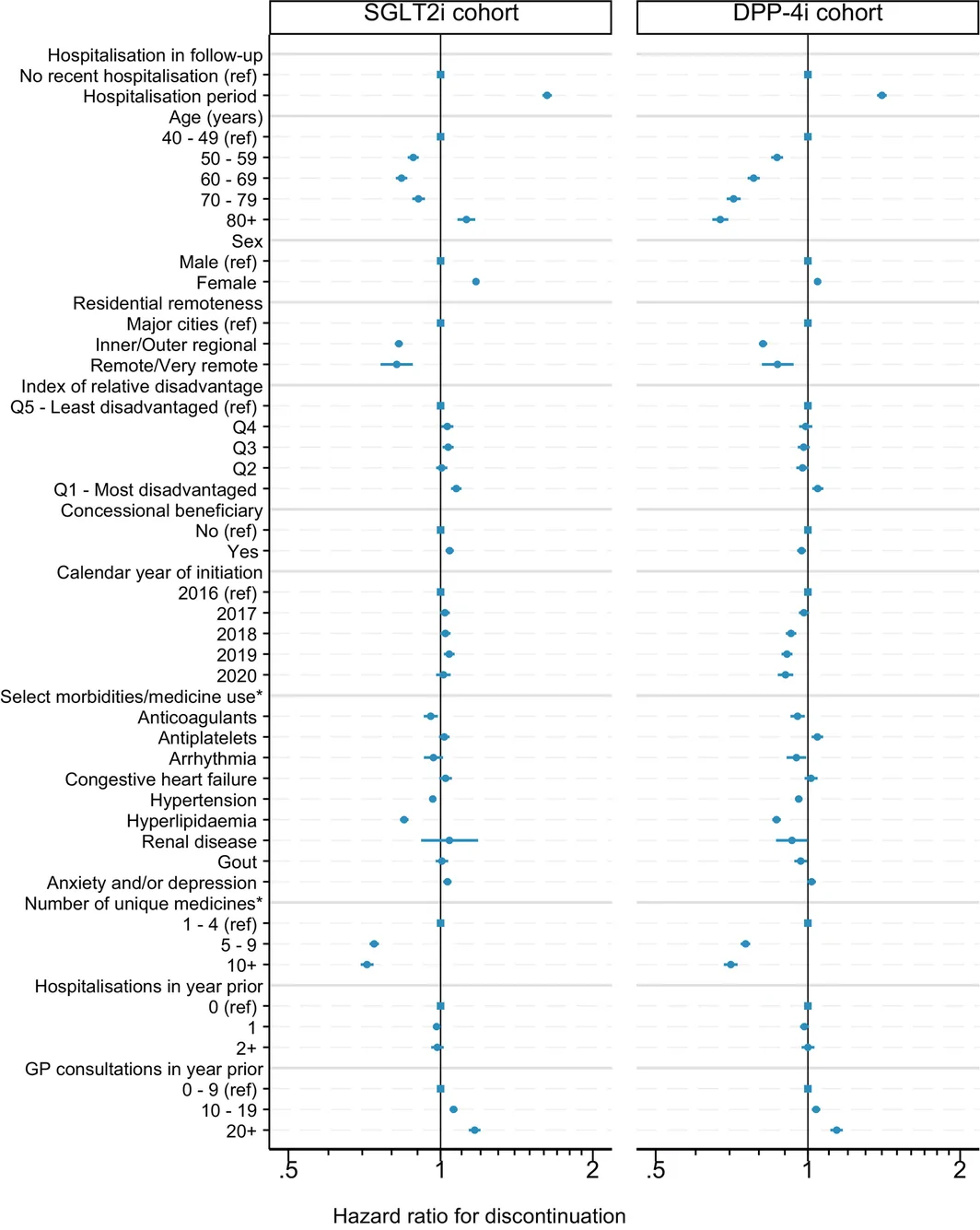
Adjusted hazard ratios for association between all‐cause hospitalisation and other characteristics, with discontinuation. *Estimated using the Rx‐Risk Comorbidity Index.

In DPP‐4i‐treated people, the adjusted risk of DPP‐4i discontinuation was 1.5 times higher (95% CI: 1.51–1.58) in the hospitalisation period compared to other times. Similar to SGLT2i, hospitalisation was one of the strongest risk factors for DPP‐4i discontinuation compared to other variables measured (Figure 1 and Table S4).

### 3.4 Sensitivity Analysis

In SGLT2i‐treated people, we found 6388 (6.0%) initiated a GLP‐1 RA at the time SGLT2i were discontinued. When these people were censored, the adjusted risk of SGLT2i discontinuation was 1.9 times higher (HR: 1.88; 95% CI: 1.83–1.92) during a hospitalisation period compared to other times.

In DPP‐4i‐treated people, 16 174 (17.6%) initiated an SGLT2i or GLP‐1 RA in the period used to define discontinuation. When these individuals were censored, the adjusted risk of DPP‐4i discontinuation was 1.7 times higher (HR: 1.69, 95% CI: 1.64–1.74) in the hospitalisation period compared to other times.

Hence, for both cohorts, adjusting for a potential change to an anti‐hyperglycaemic medicine with cardiorenal protection did not weaken the adjusted association between all‐cause hospitalisation and discontinuation of the medicine of interest.

When we changed the definition of a ‘hospitalisation period’ as the hospital stay plus 30 days, rather than 90 days following discharge, there was a stronger association between hospitalisation and discontinuation (Table S5). In SGLT2i‐treated people, the adjusted risk of SGLT2i discontinuation was 2.3 times higher (HR: 2.34, 95% CI: 2.28–2.40) in the hospitalisation period compared to other times. In DPP‐4i‐treated people, the adjusted risk of DPP‐4i discontinuation was 1.9 times higher (HR: 1.89, 95% CI: 1.84–1.95) in the hospitalisation period compared to other times.

## 4 Discussion

We found hospitalisation to be a strong risk factor for SGLT2i discontinuation with an 80% higher adjusted risk of discontinuation compared with non‐hospitalised periods. Notably, hospitalisation was also a risk factor for DPP‐4i discontinuation despite the absence of inpatient safety concerns. The higher risk of post‐hospitalisation discontinuation of SGLT2i and DPP‐4i was not explained by switching to alternative cardioprotective therapies. Our findings were strengthened when we shortened the hospitalisation period to 30 days, rather than 90 days following discharge highlighting the close relationship between hospitalisation and discontinuation. These results signify the need for hospital systems to recognise hospital admissions as a high‐risk event for medicine discontinuation for people with type 2 diabetes and to adopt strategies to minimise unwarranted SGLT2i discontinuation on discharge.

This is one of the first whole‐of‐population studies to examine the risk of SGLT2i discontinuation following hospitalisation. Prior research is limited. A United States study of veterans with advanced CKD reported higher rates of SGLT2i discontinuation during a period of hospitalisation, but not in the 60 day post‐discharge period [10]. Other studies have focused on demographic and clinical risk factors for SGLT2i discontinuation without examining hospitalisation as a time‐varying exposure [11, 12]. Our findings are consistent with the hypothesis that SGLT2i are frequently withheld in hospital and not restarted at discharge [13], reflecting historical guidance to pause therapy because of the risk of DKA [14]. Emerging evidence challenges this approach: continuing SGLT2i during hospitalisation has been associated with lower inpatient mortality without increased acute kidney injury [15]. The American Diabetes Association guideline now recommends continuing SGLT2i use in selected hospitalised patients with HF provided no contraindications [16]. Together, these findings suggest that routine interruption of SGLT2i during hospitalisation may contribute to avoidable long‐term discontinuation.

Frameworks of medicines stewardship at transitions of care emphasise medicine reconciliation as an overarching aim [6]; our findings highlight that specifically, potential inadvertent discontinuation is a key concern. There are many potential barriers to restarting medicines following the transition from hospital to community care [6]. There may be ambiguities in clinical advice on when to restart therapy, delays in patients seeing their GP post‐discharge, and problems with documentation transfer between the hospital and community. Strategies to address these issues include inpatient continuation of SGLT2i if appropriate, the input of hospital pharmacists with regards to suggesting to the treating team to potentially restart an SGLT2i prior to/on discharge and the provision of medicine reconciliation counselling and written communications plans. Additionally, hospital electronic medication management systems can flag if an SGLT2i‐treated person does not have an SGLT2i on their discharge medication list. Furthermore, instead of advising that a patient wait until they see their GP to decide on restarting an SGLT2i, hospital medical staff should advise that the patient restart once eating and drinking reasonably well [17].

The increased risk of DPP‐4i discontinuation in the hospitalisation period was surprising. Insulin is often the preferred therapy for patients with diabetes in hospital due to increased dosing flexibility [16]; it is possible DPP‐4i may be withheld in this setting. Our study demonstrates that there are broader issues impacting post‐discharge discontinuation than the clinical advice surrounding risk of SGLT2i‐associated DKA. High rates of discontinuation have been observed in other clinical populations, such as people with atrial fibrillation admitted for myocardial infarction [18]. The impact of hospitalisation on adherence to therapy for other chronic conditions needs to be better understood.

One limitation of large observational studies is that it is very difficult to determine the reasons for SGLT2i discontinuation during the hospitalisation period. A proportion of discontinuation is likely clinically appropriate, for example, if a patient experienced DKA or a serious genitourinary infection. However, we know from other studies that a low eGFR is a risk factor for SGLT2i discontinuation [10, 11]. Temporarily withholding an SGLT2i in a patient with acute kidney impairment may be reasonable but the SGLT2i should be restarted as soon as possible due to the overwhelming evidence of renoprotective effects of this medicine class [19]. This includes patients with advanced CKD where SGLT2i can and should be continued until initiation of dialysis or transplantation [20, 21]. Further quantitative and qualitative work is needed to understand the reasons responsible for the higher risk of discontinuation with hospitalisation.

Our study is also limited by assumptions on duration of therapy and ascertaining exact dates of discontinuation. Although unlikely, we cannot exclude the possibility that a person discontinued their medicine and that contributed to their hospitalisation (reverse causation)—although this would require a very short period from dispensing to discontinuation (potentially days), and a rapid escalation of cardiovascular risk. While we used a 90 day post‐hospitalisation period to ensure exhaustion of existing medicine supply, the stronger associations in our sensitivity analysis using a 30 day window suggest the risk of discontinuation is greatest immediately following hospitalisation.

Strengths of this study include whole‐of‐population data, so the results are representative of all patients with type 2 diabetes using SGLT2i and DPP‐4i rather than a subset of the population. Also, the database contains detailed hospital records, so the exact dates of hospitalisation were known, key for accurately determining the relationship between hospitalisation and discontinuation.

In conclusion, hospitalisation is a strong risk factor for SGLT2i and DPP‐4i discontinuation. Given SGLT2i are essential medicines for people with type 2 diabetes due to cardiorenal protection with harm resulting from cessation, strategies should be devised and implemented to ensure that these medicines, when paused during admission to hospital are restarted when safe to do so. Our study also highlights the potential value in examining the associations between hospitalisation and discontinuation of other critical diabetes and non‐diabetes medicines.

## Funding

This research is supported by a NHMRC Medicines Intelligence Centre of Research Excellence (grant number: 1196900) and Ideas grant (grant number: 2002889). J.L. acknowledges funding support from The Ian Potter Foundation and University of New South Wales. B.L.N. is supported by grants from the National Health and Medical Research Council of Australia, Medical Research Future Fund, Ramaciotti Foundation, and New South Wales Health. M.O.F. is supported by a Future Leader Fellowship from the National Heart Foundation of Australia (grant number: 105609).

## Conflicts of Interest

T.Y.M. has been a speaker for a Boehringer Ingelheim talk. She has received an honorarium for a talk for Eli Lilly. C.P. has been a steering committee member for meetings for AstraZeneca, Novo Nordisk and CSL. She has been on advisory boards for AstraZeneca, NovoNordisk and Novartis, and given talks for AstraZeneca, Boehringer Ingelheim and Novartis. C.P. has received travel support from Sobi. B.L.N. has received fees for advisory boards, scientific presentations, steering committee roles, travel and publication support from AstraZeneca, Alexion, Bayer, Boehringer Ingelheim, Cornerstone Medical Education, CSL‐Behring, CSL‐Seqirus, the Limbic, Medscape, Menarini, MJH Life Sciences, Proton Intelligence, Novo Nordisk, Otsuka, Sobi, Travere, and Vera Therapeutics. M.J. is responsible for research projects that have received research funding from Boehringer Ingelheim and Eli Lilly Alliance. S.‐A.P. is a member of the Drug Utilisation Sub Committee of the Pharmaceutical Benefits Advisory Committee. The views expressed in this paper do not represent those of the Committee. The Medicines Intelligence Research Program, UNSW has received research funding from IQVIA Australia, unrelated to this project.

## Supporting information


**Figure S1:** Study design.
**Table S1:** World Health Organisation Anatomical Therapeutic Chemical codes for identifying SGLT2i, DPP‐4i available in Australia.
**Table S2:** Characteristics of SGLT2i cohort by discontinuation status.
**Table S3:** Characteristics of DPP‐4i cohort by discontinuation status.
**Table S4:** Adjusted hazard ratios for association between all‐cause hospitalisation, and other characteristics, with discontinuation.
**Table S5:** Sensitivity analysis—discontinuation rates among people treated with SGLT2i or DPP‐4i, overall and stratified by hospitalisation time period when hospitalisation period is defined as the hospitalisation stay plus 30 days, rather than 90 days following discharge.

## Data Availability

The data were provided by the Australian and NSW Governments. Access to these data by other individuals or authorities are not permitted without the express permission of the approving human research ethics committees and data custodians.

## References

[1] C. E. Ndumele , J. Rangaswami , S. L. Chow , et al., “Cardiovascular‐Kidney‐Metabolic Health: A Presidential Advisory From the American Heart Association,” Circulation 148, no. 20 (2023): 1606–1635. 10.1161/CIR.0000000000001184 37807924

[2] M. Packer , J. Butler , C. Zeller , et al., “Blinded Withdrawal of Long‐Term Randomized Treatment With Empagliflozin or Placebo in Patients With Heart Failure,” Circulation 148, no. 13 (2023): 1011–1022. 10.1161/CIRCULATIONAHA.123.065748 PMC10516173 37621153

[3] NSW Agency for Clinical Innovation , Use of SGLT2‐Inhibitors for Type 2 Diabetes in Acute Inpatient Care: Clinical Practice Guide (ACI, 2023), https://aci.health.nsw.gov.au/__data/assets/pdf_file/0010/825085/ACI‐Use‐of‐SGLT2‐inhibitor‐medicines‐for‐people‐with‐diabetes.pdf.

[4] T. Y. Milder , J. Lin , S. A. Pearson , et al., “Discontinuation of SGLT2i in People With Type 2 Diabetes Following Hospitalisation for Heart Failure: A Cause for Concern?,” Diabetes, Obesity & Metabolism 27, no. 2 (2025): 997–1000. 10.1111/dom.16061 39511848

[5] M. E. Malik , A. C. Falkentoft , J. Jensen , et al., “Discontinuation and Reinitiation of SGLT‐2 Inhibitors and GLP‐1R Agonists in Patients With Type 2 Diabetes: A Nationwide Study From 2013 to 2021,” Lancet Regional Health Europe 29 (2023): 100617. 10.1016/j.lanepe.2023.100617 PMC10230617 37265783

[6] Australian Commission on Safety and Quality in Health Care , Medication Management at Transitions of Care Stewardship Framework (ACSQHC, 2025), https://www.safetyandquality.gov.au/sites/default/files/resources/attachments/medication‐management‐at‐transitions‐of‐care‐stewardship‐framework.pdf.

[7] The Commonwealth Fund , “International Health Care System Profiles: Australia,” 2026, https://www.commonwealthfund.org/international‐health‐policy‐center/countries/australia.

[8] H. Zoega , M. O. Falster , M. B. Gillies , et al., “The Medicines Intelligence Data Platform: A Population‐Based Data Resource From New South Wales, Australia,” Pharmacoepidemiology and Drug Safety 33, no. 8 (2024): e5887. 10.1002/pds.5887 39145404

[9] N. L. Pratt , M. Kerr , J. D. Barratt , et al., “The Validity of the Rx‐Risk Comorbidity Index Using Medicines Mapped to the Anatomical Therapeutic Chemical (ATC) Classification System,” BMJ Open 8, no. 4 (2018): e021122. 10.1136/bmjopen-2017-021122 PMC5905736 29654048

[10] L. P. Gregg , P. A. Richardson , V. Nambi , et al., “Sodium‐Glucose Cotransporter‐2 Inhibitor and Glucagon‐Like Peptide‐1 Receptor Agonist Discontinuation in Patients With CKD,” Journal of the American Society of Nephrology 36, no. 1 (2025): 87–98. 10.1681/ASN.0000000000000477 PMC11706553 39186372

[11] W. Alkabbani , B. R. Shah , A. Zongo , D. T. Eurich , M. W. Alsabbagh , and J. M. Gamble , “Post‐Initiation Predictors of Discontinuation of the Sodium‐Glucose Cotransporter‐2 Inhibitors: A Comparative Cohort Study From the United Kingdom,” Diabetes, Obesity & Metabolism 25, no. 12 (2023): 3490–3500. 10.1111/dom.15241 37563767

[12] C. E. Lim , B. Pasternak , B. Eliasson , and P. Ueda , “Treatment Discontinuation Among Users of GLP‐1 Receptor Agonists and SGLT2 Inhibitors in a National Population of Individuals With Type 2 Diabetes,” Diabetologia 68, no. 8 (2025): 1680–1695. 10.1007/s00125-025-06439-x PMC12246030 40316732

[13] B. L. Murphy , S. Chahal , E. Shepherd , N. Taylor , and A. Camp , “Impact of Hospitalization on Continuation of SGLT2 Inhibitors and GLP‐1 Receptor Agonists for Comorbidities in Patients With Type 2 Diabetes,” Innovations in Pharmacy 15, no. 4 (2024): 9. 10.24926/iip.v15i4.6432 PMC12090087 40401295

[14] American Diabetes Association , “16. Diabetes Care in the Hospital: Standards of Care in Diabetes‐2023,” Diabetes Care 46, no. Suppl 1 (2023): S267–S278. 10.2337/dc23-S016 PMC9810470 36507644

[15] L. G. Singh , S. Ntelis , T. Siddiqui , S. L. Seliger , J. D. Sorkin , and E. K. Spanakis , “Association of Continued Use of SGLT2 Inhibitors From the Ambulatory to Inpatient Setting With Hospital Outcomes in Patients With Diabetes: A Nationwide Cohort Study,” Diabetes Care 47, no. 6 (2024): 933–940. 10.2337/dc23-1129 PMC11294633 38051789

[16] American Diabetes Association , “16. Diabetes Care in the Hospital: Standards of Care in Diabetes‐2026,” Diabetes Care 49, no. Suppl 1 (2026): S339–S355. 10.2337/dc26-S016 PMC12690180 41358892

[17] Council of Australian Therapeutic Advisory Groups , Perioperative Management of SGLT2 Inhibitors and GLP‐1 Receptor Agonists: Practice Tool (CATAG, 2024), https://catag.org.au/wp‐content/uploads/CATAG‐Topic_Diabetes_Practice‐Tool_F.pdf.

[18] C. T. Deakin , J. D. O. Costa , D. Brieger , et al., “Post‐Discharge Pharmacotherapy in People With Atrial Fibrillation Hospitalized for Acute Myocardial Infarction: An Australian Cohort Study 2018‐22,” European Heart Journal ‐ Quality of Care and Clinical Outcomes 11, no. 3 (2025): 259–270. 10.1093/ehjqcco/qcae068 39118377

[19] B. L. Neuen , R. A. Fletcher , S. D. Anker , et al., “SGLT2 Inhibitors and Kidney Outcomes by Glomerular Filtration Rate and Albuminuria: A Meta‐Analysis,” Journal of the American Medical Association 335, no. 3 (2026): 233–244. 10.1001/jama.2025.20834 PMC12595549 41203232

[20] Kidney Disease: Improving Global Outcomes (KDIGO) CKD Work Group , “KDIGO 2024 Clinical Practice Guideline for the Evaluation and Management of Chronic Kidney Disease,” Kidney International 105, no. 4S (2024): S117–S314. 10.1016/j.kint.2023.10.018 38490803

[21] S. Chatur , R. A. Fletcher , E. Yeung , et al., “SGLT2 Inhibition in Patients With Type 2 Diabetes and CKD Experiencing a Deterioration in Estimated Glomerular Filtration Rate to < 20ml/min/1.73m^2^ ,” Journal of Cardiac Failure 32, no. 8 (2026): 1408–1417, 10.1016/j.cardfail.2026.01.020. 41765341

