# Supplementary material for: Hospitalisation Is a Strong Risk Factor for Discontinuation of Sodium‐Glucose Cotransporter 2 Inhibitors for Type 2 Diabetes
Source: Diabetes Obes Metab. 2026 Aug 17;28(11):10210–7. doi: 10.1111/dom.71219 (PMC13646384; doi:10.1111/dom.71219)
Supplement: Supplementary file 1 [file DOM-28-10210-s001.docx]

**Supplementary Figure 1.** Study design

**Supplementary Table 1.** World Health Organization Anatomical Therapeutic Chemical codes for identifying SGLT2i, DPP-4i available in Australia

| **Medicine** | **Anatomical Therapeutic Chemical codes** |
| --- | --- |
| **SGLT2i** |  |
| Dapagliflozin and metformin | A10BD15 |
| Empagliflozin and linagliptin | A10BD19 |
| Empagliflozin and metformin | A10BD20 |
| Saxagliptin and dapagliflozin | A10BD21 |
| Ertugliflozin and metformin | A10BD23 |
| Ertugliflozin and sitagliptin | A10BD24 |
| Dapagliflozin | A10BK01 |
| Empagliflozin | A10BK03 |
| Ertugliflozin | A10BK04 |
| Canagliflozin | A10BX11 |
|  |  |
| **DPP-4i** |  |
| Sitagliptin and metformin | A10BD07 |
| Vildagliptin and metformin | A10BD08 |
| Saxagliptin and metformin | A10BD10 |
| Linagliptin and metformin | A10BD11 |
| Alogliptin and metformin | A10BD13 |
| Empagliflozin and linagliptin | A10BD19 |
| Saxagliptin and dapagliflozin | A10BD21 |
| Ertugliflozin and sitagliptin | A10BD24 |
| Sitagliptin | A10BH01 |
| Vildagliptin | A10BH02 |
| Saxagliptin | A10BH03 |
| Alogliptin | A10BH04 |
| Linagliptin | A10BH05 |
| Sitagliptin and simvastatin | A10BH51 |

Note: Canagliflozin was only available in Australia between December 2013 and August 2015

**Supplementary Table 2.** Characteristics of SGLT2i cohort by discontinuation status

| Characteristics | Discontinuation | No discontinuation |
| --- | --- | --- |
|  | N=66,989 | N=39,109 |
| Age, years, median (IQR) | 63 (55-71) | 64 (56-71) |
| Age categories, years, n (%) |  |  |
| 40-49 | 9,593 (14.3) | 4,133 (10.6) |
| 50-59 | 17,764 (26.5) | 10,050 (25.7) |
| 60-69 | 21,627 (32.3) | 14,183 (36.3) |
| 70-79 | 14,249 (21.3) | 8,938 (22.9) |
| 80+ | 3,756 (5.6) | 1,805 (4.6) |
| Sex, n (%) |  |  |
| Male | 38,979 (58.2) | 25,250 (64.6) |
| Female | 28,010 (41.8) | 13,859 (35.4) |
| Residence remoteness, n (%) |  |  |
| Major cities | 47,174 (70.4) | 24,719 (63.2) |
| Inner regional/outer regional | 18,716 (27.9) | 13,662 (34.9) |
| Remote/very remote | 745 (1.1) | 573 (1.5) |
| Unknown | 354 (0.5) | 155 (0.4) |
| Socioeconomic status, n (%) |  |  |
| Q5 (least disadvantaged) | 12,351 (18.4) | 7,026 (18.0) |
| Q4 | 8,770 (13.1) | 4,856 (12.4) |
| Q3 | 13,782 (20.6) | 8,175 (20.9) |
| Q2 | 14,261 (21.3) | 9,213 (23.6) |
| Q1 (most disadvantaged) | 17,465 (26.1) | 9,683 (24.8) |
| Unknown | 360 (0.5) | 156 (0.4) |
| Concessional beneficiaries, n (%) | 38,899 (58.1) | 22,629 (57.9) |
| Calendar year of SGLT2i initiation, n (%) |  |  |
| 2016 | 14,167 (21.1) | 5,497 (14.1) |
| 2017 | 18,801 (28.1) | 8,543 (21.8) |
| 2018 | 15,730 (23.5) | 9,091 (23.2) |
| 2019 | 13,068 (19.5) | 10,220 (26.1) |
| 2020 | 5,223 (7.8) | 5,758 (14.7) |
| History of selected morbidities and medicine use†, n (%): |  |  |
| Anticoagulants | 5,960 (8.9) | 3,918 (10.0) |
| Antiplatelets | 8,958 (13.4) | 5,256 (13.4) |
| Arrhythmia | 2,865 (4.3) | 1,883 (4.8) |
| Congestive heart failure | 6,211 (9.3) | 3,815 (9.8) |
| Hypertension | 29,179 (43.6) | 18,038 (46.1) |
| Hyperlipidaemia | 49,988 (74.6) | 31,505 (80.6) |
| Gout | 5,727 (8.5) | 3,779 (9.7) |
| Renal disease | 231 (0.3) | 121 (0.3) |
| Anxiety and/or depression | 17,490 (26.1) | 9,890 (25.3) |
| Number of unique medicines dispensed in 90 days prior to and including the index date, n (%) |  |  |
| 1-4 | 18,265 (27.3) | 7,913 (20.2) |
| 5-9 | 34,022 (50.8) | 22,609 (57.8) |
| 10+ | 14,702 (21.9) | 8,587 (22.0) |
| Number of hospitalisations in year prior to and including the index date, n (%) |  |  |
| 0 | 42,127 (62.9) | 24,973 (63.9) |
| 1 | 18,473 (27.6) | 10,761 (27.5) |
| >1 | 6,389 (9.5) | 3,375 (8.6) |
| Number of GP consultations in year prior to index date, n (%) |  |  |
| 0-9 | 36,436 (54.4) | 22,565 (57.7) |
| 10-19 | 22,473 (33.5) | 12,732 (32.6) |
| 20+ | 8,080 (12.1) | 3,812 (9.7) |

†Estimated using the Rx-Risk Comorbidity Index

Abbreviation: IQR interquartile range

**Supplementary Table 3.** Characteristics of DPP-4i cohort by discontinuation status

| Characteristics | Discontinuation | No discontinuation |
| --- | --- | --- |
|  | N=58,572 | N=33,388 |
| Age, years, median (IQR) | 64 (55-73) | 68 (60-76) |
| Age categories, years, n (%) |  |  |
| 40-49 | 8,148 (13.9) | 2,538 (7.6) |
| 50-59 | 14,120 (24.1) | 6,102 (18.3) |
| 60-69 | 17,344 (29.6) | 9,916 (29.7) |
| 70-79 | 12,936 (22.1) | 9,436 (28.3) |
| 80+ | 6,024 (10.3) | 5,396 (16.2) |
| Sex, n (%) |  |  |
| Male | 33,371 (57.0) | 19,203 (57.5) |
| Female | 25,201 (43.0) | 14,185 (42.5) |
| Residence remoteness, n (%) |  |  |
| Major cities | 42,119 (71.9) | 21,519 (64.5) |
| Inner regional/outer regional | 15,391 (26.3) | 11,282 (33.8) |
| Remote/very remote | 767 (1.3) | 463 (1.4) |
| Unknown | 295 (0.5) | 124 (0.4) |
| Socioeconomic status, n (%) |  |  |
| Q5 (least disadvantaged) | 10,406 (17.8) | 5,618 (16.8) |
| Q4 | 7,580 (12.9) | 4,159 (12.5) |
| Q3 | 11,734 (20.0) | 7,054 (21.1) |
| Q2 | 12,349 (21.1) | 7,764 (23.3) |
| Q1 (most disadvantaged) | 16,203 (27.7) | 8,666 (26.0) |
| Unknown | 300 (0.5) | 127 (0.4) |
| Concessional beneficiaries, n (%) | 35,848 (61.2) | 23,167 (69.4) |
| Calendar year of DPP-4i initiation, n (%) |  |  |
| 2016 | 15,736 (26.9) | 5,279 (15.8) |
| 2017 | 14,243 (24.3) | 6,172 (18.5) |
| 2018 | 13,583 (23.2) | 8,079 (24.2) |
| 2019 | 10,965 (18.7) | 9,156 (27.4) |
| 2020 | 4,045 (6.9) | 4,702 (14.1) |
| History of selected morbidities and medicine use†, n (%): |  |  |
| Anticoagulants | 5,894 (10.1) | 4,765 (14.3) |
| Antiplatelets | 7,524 (12.8) | 4,886 (14.6) |
| Arrhythmia | 2,729 (4.7) | 2,285 (6.8) |
| Congestive heart failure | 6,081 (10.4) | 4,557 (13.6) |
| Hypertension | 24,812 (42.4) | 16,239 (48.6) |
| Hyperlipidaemia | 41,052 (70.1) | 25,890 (77.5) |
| Gout | 5,385 (9.2) | 3,916 (11.7) |
| Renal disease | 773 (1.3) | 560 (1.7) |
| Anxiety and/or depression | 14,930 (25.5) | 9,159 (27.4) |
| Number of unique medicines dispensed in 90 days prior to and including the index date, n (%) |  |  |
| 1-4 | 17,933 (30.6) | 6,382 (19.1) |
| 5-9 | 27,709 (47.3) | 17,586 (52.7) |
| 10+ | 12,930 (22.1) | 9,420 (28.2) |
| Number of hospitalisations in year prior to and including the index date, n (%) |  |  |
| 0 | 35,502 (60.6) | 19,073 (57.1) |
| 1 | 16,580 (28.3) | 10,169 (30.5) |
| >1 | 6,490 (11.1) | 4,146 (12.4) |
| Number of GP consultations in year prior to index date, n (%) |  |  |
| 0-9 | 32,283 (55.1) | 17,824 (53.4) |
| 10-19 | 18,894 (32.3) | 11,403 (34.2) |
| 20+ | 7,395 (12.6) | 4,161 (12.5) |

† Estimated using the Rx-Risk Comorbidity Index

Abbreviation: IQR interquartile range

**Supplementary Table 4.** Adjusted hazard ratios for association between all-cause hospitalisation, and other characteristics, with discontinuation

| Characteristics | SGLT2i cohort –  SGLT2i discontinuation | | DPP-4i cohort –  DPP-4i discontinuation | |
| --- | --- | --- | --- | --- |
|  | HR (95% CI) | P-value | HR (95% CI) | P-value |
| All-cause hospitalisation during follow-up |  | <0.001 |  | <0.001 |
| No | Ref |  | Ref |  |
| Yes | 1.83 (1.79, 1.87) |  | 1.55 (1.51, 1.58) |  |
| Age categories, years |  | <0.001 |  | <0.001 |
| 40-49 | Ref |  | Ref |  |
| 50-59 | 0.88 (0.86, 0.90) |  | 0.87 (0.85, 0.89) |  |
| 60-69 | 0.83 (0.81, 0.85) |  | 0.78 (0.76, 0.80) |  |
| 70-79 | 0.89 (0.87, 0.92) |  | 0.71 (0.69, 0.73) |  |
| 80+ | 1.12 (1.07, 1.16) |  | 0.67 (0.65, 0.70) |  |
| Sex |  | <0.001 |  | <0.001 |
| Male | Ref |  | Ref |  |
| Female | 1.17 (1.16, 1.19) |  | 1.05 (1.03, 1.06) |  |
| Residence remoteness |  | <0.001 |  | <0.001 |
| Major cities | Ref |  | Ref |  |
| Inner regional/outer regional | 0.83 (0.81, 0.84) |  | 0.81 (0.80, 0.83) |  |
| Remote/very remote | 0.82 (0.76, 0.88) |  | 0.87 (0.81, 0.93) |  |
| Unknown | 0.43 (0.19, 0.96) |  | 1.12 (0.46, 2.72) |  |
| Socioeconomic status |  | <0.001 |  | <0.001 |
| Q5 (least disadvantaged) | Ref |  | Ref |  |
| Q4 | 1.03 (1.00, 1.06) |  | 0.99 (0.96, 1.02) |  |
| Q3 | 1.03 (1.01, 1.06) |  | 0.98 (0.96, 1.01) |  |
| Q2 | 1.01 (0.98, 1.03) |  | 0.98 (0.95, 1.00) |  |
| Q1 (most disadvantaged) | 1.07 (1.05, 1.10) |  | 1.05 (1.02, 1.07) |  |
| Unknown | 2.50 (1.12, 5.56) |  | 0.95 (0.40, 2.29) |  |
| Concessional beneficiaries, n (%) |  | <0.001 |  | 0.005 |
| No | Ref |  | Ref |  |
| Yes | 1.04 (1.02, 1.06) |  | 0.97 (0.95, 0.99) |  |
| Calendar year of SGLT2i/DPP-4i initiation |  | 0.015 |  | <0.001 |
| 2016 | Ref |  | Ref |  |
| 2017 | 1.02 (1.00, 1.05) |  | 0.98 (0.96, 1.01) |  |
| 2018 | 1.03 (1.00, 1.05) |  | 0.93 (0.91, 0.95) |  |
| 2019 | 1.04 (1.02, 1.07) |  | 0.91 (0.89, 0.94) |  |
| 2020 | 1.02 (0.98, 1.05) |  | 0.91 (0.87, 0.94) |  |
| History of selected morbidities and medicine use† | Ref is no use |  | Ref is no use |  |
| Anticoagulants | 0.96 (0.93, 0.99) | 0.007 | 0.96 (0.93, 0.99) | 0.012 |
| Antiplatelets | 1.02 (1.00, 1.05) | 0.099 | 1.05 (1.02, 1.08) | <0.001 |
| Arrhythmia | 0.97 (0.93, 1.01) | 0.161 | 0.95 (0.91, 0.99) | 0.014 |
| Congestive heart failure | 1.03 (1.00, 1.06) | 0.033 | 1.02 (0.99, 1.05) | 0.211 |
| Hypertension | 0.96 (0.95, 0.98) | <0.001 | 0.96 (0.94, 0.98) | <0.001 |
| Hyperlipidaemia | 0.85 (0.83, 0.86) | <0.001 | 0.86 (0.85, 0.88) | <0.001 |
| Gout | 1.00 (0.97, 1.03) | 0.890 | 0.97 (0.94, 0.99) | 0.016 |
| Renal disease | 1.05 (0.92, 1.19) | 0.497 | 0.98 (0.92, 1.06) | 0.663 |
| Anxiety and/or depression | 1.03 (1.01, 1.05) | 0.003 | 1.02 (1.00, 1.04) | 0.147 |
| Number of unique medicines dispensed in 90 days prior to and including the index date |  | <0.001 |  | <0.001 |
| 1-4 | Ref |  | Ref |  |
| 5-9 | 0.74 (0.73, 0.76) |  | 0.76 (0.74, 0.77) |  |
| 10+ | 0.72 (0.70, 0.75) |  | 0.71 (0.69, 0.74) |  |
| Number of hospitalisations in year prior to and including the index date |  | <0.001 |  | <0.001 |
| 0 | Ref |  | Ref |  |
| 1 | 1.01 (0.99, 1.03) |  | 1.01 (0.99, 1.03) |  |
| >1 | 1.06 (1.03, 1.09) |  | 1.06 (1.03, 1.09) |  |
| Number of GP consultations in year prior to index date |  | <0.001 |  | <0.001 |
| 0-9 | Ref |  | Ref |  |
| 10-19 | 1.05 (1.04, 1.07) |  | 1.03 (1.01, 1.05) |  |
| 20+ | 1.16 (1.13, 1.19) |  | 1.13 (1.09, 1.16) |  |

†Estimated using the Rx-Risk Comorbidity Index

Abbreviation: Ref reference

**Supplementary Table 5.** Sensitivity analysis – discontinuation rates among people treated with SGLT2i or DPP-4i, overall and stratified by hospitalisation time period when hospitalisation period is defined as the hospitalisation stay plus 30 days, rather than 90 days following discharge

|  | SGLT2i cohort | DPP-4i cohort |
| --- | --- | --- |
| Overall follow-up |  |  |
| Total person-years | 169,973 | 141,224 |
| Number discontinued | 66,989 | 58,572 |
| Rate per 100 person-years | 39.4 | 41.5 |
| During a hospitalisation period |  |  |
| Total person-years | 7,490 | 8,268 |
| Number discontinued | 6,341 | 5,576 |
| Rate per 100 person-years | 84.7 | 67.4 |
| All other follow-up times |  |  |
| Total person-years | 162,483 | 132,956 |
| Number discontinued | 60,648 | 52,996 |
| Rate per 100 person-years | 37.3 | 39.9 |
